# Dynamic Analysis of Major Components in the Different Developmental Stages of *Tenebrio molitor*

**DOI:** 10.3389/fnut.2021.689746

**Published:** 2021-09-20

**Authors:** Xiao Yu, Qiang He, Dun Wang

**Affiliations:** Institute of Entomology, Northwest A&F University, Yangling, China

**Keywords:** *Tenebrio molitor*, major components, amino acids profile, developmental stages, harvest time

## Abstract

The yellow mealworm, *Tenebrio molitor*, is an important resource insect with a high protein percentage that is widely farmed in many countries. In this study, the content dynamics for protein, fat, chitin, and other components in the whole development process of yellow mealworms were analyzed by sampling from different instars and combining with their growth conditions. The results of the component dynamic analyses in the different development stages showed that the percentages of protein, fat, and chitin were the highest in the larval stage, pupal stage, and adult stage, respectively. The results of amino acid composition dynamic analysis also indicated comparatively higher essential amino acids in the earlier instar (e.g., before the 9th instar) larvae. Therefore, we found that the earlier instar is better than the final instar as the insect farming harvest time. Furthermore, the larvae in the earlier instar consumed dramatically less feed and could effectively reduce the farming costs of insect farmers. This finding provides an alternative option to farm insects for different purposes and in an economic way.

## Introduction

*Tenebrio molitor* (Coleoptera: Tenebrionidae) is a common storage pest that is distributed throughout the world. This mealworm goes through several stages: egg, larva, pupa, and adult. Within these different stages, the mealworm morphologies vary greatly. *Tenebrio molitor* displays variability in developmental time (1, 2), as the number of instars can vary from 9 to 20 (3, 4) depending on several abiotic factors, such as temperature, humidity, oxygen concentration, and biotic factors, such as population density, parental age, and feed quality (5–10). Furthermore, the number of instars in *T. molitor* decreases in response to befitting conditions (1).

The larvae of *T. molitor* (AKA yellow mealworm) are a common protein source for livestock and pets because they are easy to breed, high in protein, and have a high rate of assimilation of nitrogen from their feed (11–14). The yellow mealworm is not only a good source of protein, for instance, the effect of feeding yellow mealworms to chickens and fish as a protein supplement for herbivores (15–18), but also beneficial for improving the utilization rate of the intestinal protein of livestock, promoting their growth, increasing yield, and optimizing quality (19).

Numerous international organizations like the Food and Agriculture Organization (FAO) have been investigating the possibility of using *T. molitor* as a protein source of human nutrition to solve malnutrition worldwide (20, 21). Meanwhile, with the COVID-19 pandemic as a background, nutritional advice has suggested that protein is important to immunity and recovery. Studies have shown that a high-protein diet can ameliorate the decrease in amino acid absorption caused by gastrointestinal disorders in patients with COVID-19 (22). Thus, an increase in the daily protein intake of patients from 0.8 to 1 g/kg (23) has been recommended. With freeze-dried yellow mealworm larvae containing approximately 50% crude protein and its protein extract having a true protein content of approximately 75%, it has been observed that mealworms are increasingly being incorporated into food either as whole insects in fresh or dried form, as rough machining products, or as crude extracts to increase their nutritional value or functionality (24).

The nutritional values of proteins are determined by their amino acid composition (25). The contents of leucine (Leu), isoleucine (Ile), arginine (Arg), glycine (Gly), and Valine (Val) are important protein indices of a rehabilitation diet. These amino acids regulate the mechanistic target of rapamycin (mTOR) pathway and contribute to the anti-inflammation and reconstruction of damaged tissues for patients (24, 26, 27). Furthermore, muscle development has been positively correlated with essential amino acid intake (28). The yellow mealworm has also been reported to contain all the essential amino acids needed for human nutrition (29), but there is still a lack of research on the nutritional characteristics of the different stages of yellow mealworms.

As far as we know, few data available on the nutritional characteristics of the different instars of *T. molitor* that contents of the protein and information on the amino acid composition together. Thus, the objective of this study was to determine the nutrient, protein, and amino acid compositions and nutritional values of *T. molitor* in relation to the different developmental stages of the same sources to search for the appropriate stage of harvest.

## Materials and Methods

### Sample Preparation

The experimental yellow mealworms were reared by the authors in the insect-breeding room of the Insect Museum of Northwest A & F University. Mealworms were raised in plastic boxes (45 cm long × 19 cm wide × 10 cm deep) and fed wheat bran with an added 25–50 g of fresh cabbage leaves or broccoli once a day. The amount of vegetables per day was adjusted according to the daily consumption of the yellow mealworms to make sure that all vegetables were consumed. The feeding conditions were controlled at 25°C with 60–70% relative humidity.

We first picked healthy individuals with similar sizes from mealworms of every instar from the 3rd instar onwards, at both the pupa and adult stages. Next, we stored the chosen mealworms at −20°C. They were then separated into 8 groups and placed in clean test tubes, with every group containing 10 mealworms from every two adjacent instars. The samples were then put at 105°C for 15 min with paper. After the stage of the destructive enzyme, the samples were put at 65°C for 24 h until the mass was stable and then weighed three times to take the average. They were put back at 65°C for 3 h and then smashed. Finally, the prepared samples were stored at −20°C.

### Feed Consumption Determination

The yellow mealworms of the different instars were fed in different plastic boxes with the same feeding conditions as indicated in Section Sample preparation. Feed was added to each box and its weight was recorded. The yellow mealworms and their feces were screened every 3 days, and the weight of the remaining food was measured until the yellow mealworms in the box entered the next stage. The feeding amount of the yellow mealworms in the different instars was measured. The percentage value of the consumption of a single larva during a certain period and the consumption of the whole larva period were used as the food consumption proportion. Feed assimilability and feed–protein conversion rate were calculated by these formulas:


$$\begin{array}{l} \text{Feed assimilability}\\ \;=\;\frac{Single\;larvae\;weight\;gained}{Single\;larvae\;feed\;consumption}\;\times 100\%\\ \text{Feed}-\text{ protein conversion rate}\\ \;=\;\frac{Single\;larvae\;crude\;peotein\;gained}{Single\;larvae\;feed\;consumption}\;\times 100\% \end{array}$$

### Crude Fats Content Determination

Crude fats were extracted using a Soxhlet extractor (Model 189101, from JuLaBo, Beijing, China) by the methods of Smith and Tschinkel (30). Small holes were punctured at the top and bottom of the gelatin capsules (Model 70116, from HEAD Biotechnology, Beijing, China). Then, the sample was contained in gelatin capsules held in place with wires. The flat-bottomed flask, Soxhlet extractor, and condenser were mounted from bottom to top. The ethyl ether was added to the flask until it was two-thirds filled, and the sample-filled gelatin capsules were placed into the Soxhlet extractor. The hotplate was then heated to more than 37°C and ran for 24 h. Crude fat mass was determined by the difference between dry and lean mass.

### Chitin Content Determination

Chitin was extracted by the methods of Wang et al. (31). The dried mealworms were weighed (W_P_) and then treated with 3% HCl (v/v) for 24 h to demineralize them. After demineralization, the samples were submerged using 10% NaOH (m/v) at 100°C for 5 h to remove the protein and fat. They were then washed with tap water. The decolorization was performed using a treatment with 5% KMnO_4_ (m/v) water solution for 5 min and washed with tap water, followed by a treatment of 10% oxalic acid (m/v) water solution. The chitin was then dehydrated and weighed (W_C_). Chitin content was calculated by the formula:


$$\begin{array}{l} \text{Chitin content }=W_{C}/W_{P}\times 100\% \end{array}$$

### P and Ca Determination

The contents of phosphorus (P) and calcium (Ca) were determined by the method of McQuaker et al. (32). The dry mealworms were homogenized, and a 1 g sample was then digested in the presence of successive 15 and 10 ml aliquots of HNO_3_. After each addition, the sample was taken to near dryness. Then, 5.5 m HCIO_4_ was added, and the sample digested until dense white fumes of HCIO_4_ appeared. A simultaneous multi-element analysis was subsequently performed using the atomic absorption spectrometer (Model: ZEEnit 700P, AnalytikJena, Germany).

All results were obtained as the average of three replicates (1 g of dry powder of mealworms per replication).

### Crude Protein Determination

The mass of crude protein was determined by the Kjeldahl method (33) and a nitrogen-to-protein conversion factor of 5.41 was used (34).

### Amino Acid Analysis

The samples was pretreated with HCl (6 mol/L) at 110°C for 22 h with N_2_ for determination of Trpand Cys by alkaline hydrolysis and performic acid oxidation (35, 36), respectively. After hydrolysis, the hydrolysate was dehydrated under a vacuum, solved with sodium citrate (PH = 2.2), and then filtered through by a 0.45-mm Millipore membrane filter. The amino acid content was determined by an Amino Acid Analyzer (Beckman 121 Mb, Beckman Coulter, Inc., Chaska, MN, USA). The Essential Amino Acid Index (EAAI) was calculated using the method of Oser (37), in which reference protein data was obtained from the US Department of Agriculture (38), amino acid scores for the diverse developmental stages of people were calculated using the method of Sarana et al. (39), and the reference pattern was determined by FAO/WHO (40).

### Statistical Analysis

Data were collected from three independent replications and were expressed in terms of mean ± SD. The regression analysis of body weight changes at the larval stage was performed using Microsoft Excel (version 2019). Results were expressed as mean ± SD. Multigroup comparisons of the means were carried out by a one-way analysis ANOVA test using SPSS (version 22) with *post-hoc* contrasts by the Student–Newman–Keuls test. The statistical significance for all the tests was set at *p* < 0.05. Heating maps were drawn by Origin (version 9.6).

## Results and Discussion

### Incremental Analysis

**Figure 1:**
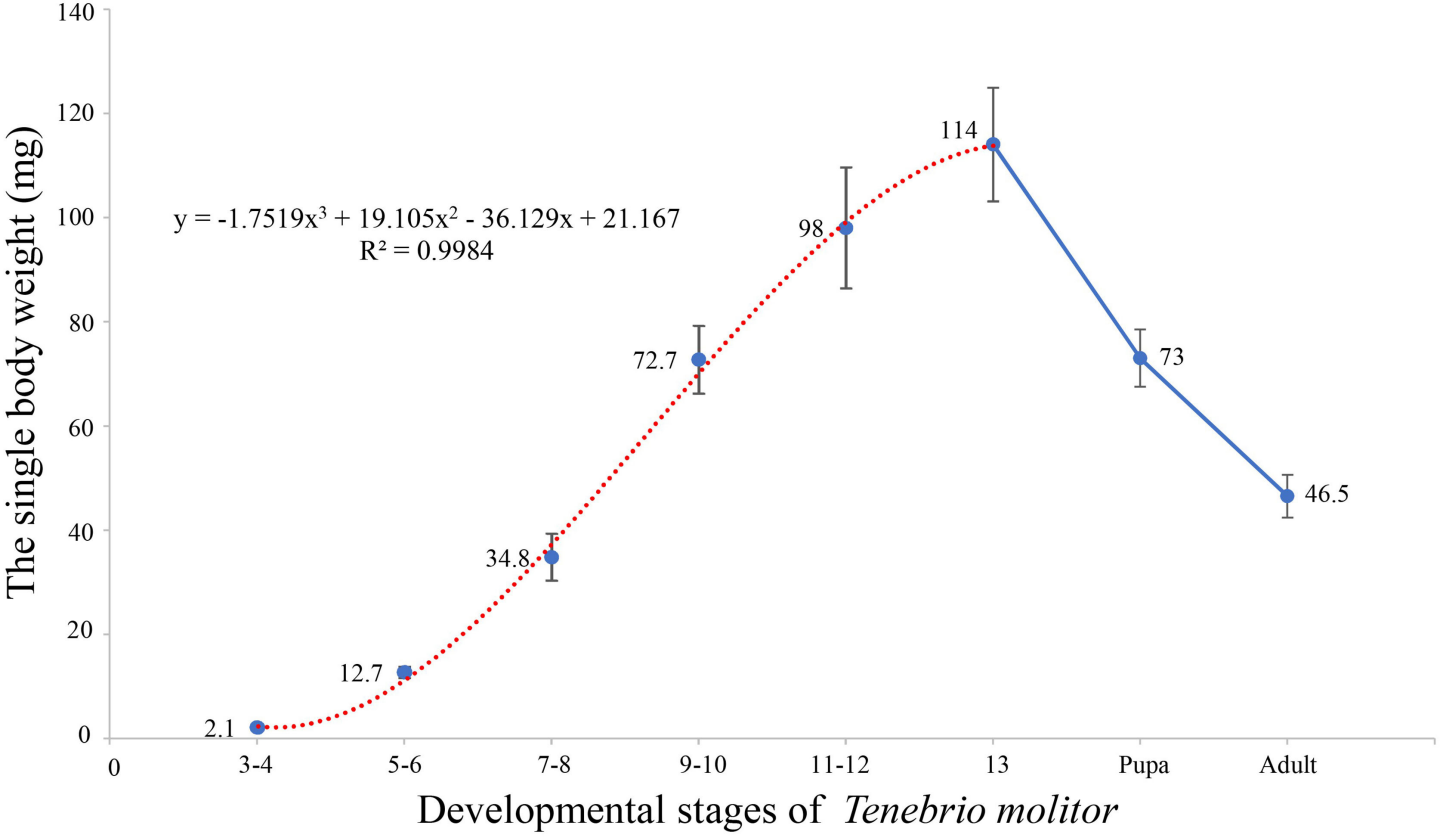
The dynamic of weight of single *Tenebrio molitor* in different stages.

The mealworms of this experiment had 13 instars, while the individual weights of the yellow mealworms increased with the growth of the instars during the larval stage (Figure 1). Fitting equation of insect weight changes in the whole larval stage are as follows:


$$\begin{array}{l} \text{y }=\;-1.7519{\text{x}}^{3}+19.105{\text{x}}^{2}-36.129\text{x}+21.167\;\left(\text{R }=\;0.978\right) \end{array}$$

According to the derivation of the formula, the growth rate of the larvae was the fastest at the 7th to 10th instars and the weight of the larvae reached its maximum at the 13th instar. In the pupal and adult stages, the weight of single larvae decreased significantly compared with that of mature larvae, which was related to changes in the main components at the different stages of the larvae.

### Proximate Analysis

The proximate analysis on a dry basis is given in Table 1. The main components were crude protein, fat, and chitin in *T. molitor*. The percentage of fat in dry weight increased from 30.4 to 36.6% before the adult phase and reached its maximum at the pupa stage. The percentage of chitin in dry weight increased from 7.2 to 11.8% with development. The phosphorus content decreased slightly with the growth of the larvae, but calcium content increased before the pupal phase. The crude protein contents of the single larvae increased with the increase of larvae weight and reached their maximum value of 114 mg at the 13th instar (Table 2). The crude protein content in the pupal stage and adult stage decreased significantly. From larval stages to pupation and adult, the percentage of crude protein decreased from 59.2 to 39.6%. Furthermore, the content of the larval stage was higher than that of the pupal and adult stages, with the crude protein content of three to four instar larvae being the highest.

**Table 1:** Proximate analysis of *Tenebrio molitor* on a dry basis (mg/g, mean ± SD).

**Nutrition component**	**Stages of development**							
	**Larvae instars**							
	**3–4**	**5–6**	**7–8**	**9–10**	**11–12**	**13**	**Pupa**	**Adult**
Crude protein	591.9 ± 10.9^a^	580.5 ± 14.4^a^	580.8 ± 17.0^ab^	555.3 ± 9.9^b^	490.2 ± 20.4^c^	486.1 ± 19.0^c^	451.5 ± 23.3^c^	395.9 ± 15.2^d^
Fat	304.1 ± 6.3^c^	302.4 ± 5.5^c^	319.5 ± 8.2^b^	333.8 ± 10.7^b^	351.0± 9.1^ab^	359.0 ± 11.3^a^	366.3 ± 14.4^a^	133.1 ± 10.7^d^
Chitin	71.9 ± 5.4^e^	84.5 ± 2.3^d^	92.6 ± 1.5^c^	92.3 ± 1.8^c^	101.3 ± 4.0^b^	99.3 ± 3.3^b^	95.4 ± 2.0^bc^	117.9 ± 1.4^a^
Ca	19.9 ± 1.0^c^	21.2 ± 0.3^c^	20.9 ± 0.9^c^	26.1 ± 1.0^b^	28.1 ± 1.2^ab^	29.3 ± 0.8^a^	27.1 ± 0.5^b^	27.2 ± 1.3^ab^
P	11.6 ± 0.1^bc^	11.0 ± 0.0^c^	12.7 ± 0.3^a^	11.6 ± 0.2^bc^	10.5± 1.0^bc^	10.4 ± 0.4^c^	10.7 ± 0.5^c^	9.3 ± 0.9^c^

*Data are presented as the mean ± SD. Means with different superscripts in the column differ significantly (p ≤ 0.05). n = 3*.

**Table 2:** The single larvae weight, feed consumption, and percentage of crude protein for the different development stages of *Tenebrio molitor*.

	**Stages of development**							
**Amount**	**Larvae instars**							
	**3–4**	**5–6**	**7–8**	**9–10**	**11–12**	**13**	**Pupa**	**Adult**
Single larvae weight (mg)	2.1 ± 0.1^f^	12.7 ± 1.1^e^	34.8 ± 4.5^d^	72.7 ± 6.5^b^	98.0 ± 11.6^a^	114 ± 10.9^a^	73.0 ± 5.5^b^	46.5 ± 4.1^c^
Crude protein weight (mg)	1.24 ± 0.1^f^	7.37 ± 0.4^e^	20.2 ± 1.8^d^	40.4 ± 5.2^b^	48.0 ± 4.4^ab^	55.3 ± 2.9^a^	33.0 ± 1.9^c^	18.4 ± 1.2^d^
Crude protein proportion (%)	59.19	58.05	58.08	55.53	49.02	48.6	45.15	39.59
Single larvae feed consumption (mg)	17.3 ± 1.1^f^	36.3 ± 2.1^e^	88.7 ± 0.7^d^	200.7 ± 5.2^c^	214.0 ± 7.9^b^	236.67 ± 9.7^a^	–	–
Feed consume proportion (%)	2.18	4.58	11.17	25.28	26.96	29.82	–	–
Feed assimilability (%)	61.26	60.83	42.74	12.61	7.48	–17.32	–	–
Feed–protein conversion rate (%)	35.43	35.31	22.78	3.79	3.41	–9.42	–	–

*Data are presented as the mean ± SD. Means with different superscripts in column differ significantly (p ≤ 0.05). n = 3*.

### Assimilation Ratio Analysis

The feed consumption and assimilation ratio analyses of single larvae is given in Table 2. The feed intake of yellow mealworms increased significantly after the 8th instar, which was the highest of the 9th to 13th instars, accounting for 82.07% of the total. In contrast, the assimilation rate and protein conversion rate of the latter phases decreased significantly, with the rate of feed assimilation of yellow mealworm falling to 7.48–12.61%. In particular, the 13th instar of the yellow mealworms had the largest feed intake; however, as they were about to enter the pupal stage, both the weight and the protein content in the body of these samples decreased instead of increasing, so the assimilation rate and protein conversion rate were negative.

### Amino Acid Analysis

The mealworms of the different periods all contained 18 amino acids, which could be measured, including eight essential amino acids (Table 3). The contents of glutamic, aspartic acid, and alanine were the highest, while the contents of tryptophan, cysteine, and methionine were the lowest. The highest contents of essential amino acids were Val, Lys, and Leu, which are involved in muscle development, signal transduction, and energy supply (26, 28, 41, 42). Among the non-essential amino acids, Glu, an important component of nitrogen metabolism, had the highest content (43). The four non-essential amino acids (glutamic acid, aspartic acid, glycine, and alanine) related to flavor accounted for approximately 20% of the dry weight of *T. molitor*, which were beneficial to the development and production of the flavorings derived from the protein of the yellow mealworm. The content of the amino acids decreased with the development of the worms, and the contents of amino acids in the pupal and adult stages as significantly lower than that in the larval stage. Thus, it can be concluded that the pupal and adult stages of *T. molitor* are not good sources of protein. In addition, the adult mealworms cannot be eaten because they contain certain toxic compounds and secretions with a strange smell (44), which is in line with the harvest situation in the production of yellow mealworms.

**Table 3:** Amino acid content at different development stages of *Tenebrio molitor* (mg/g, dry matter basis).

**Components**	**Larvae instar**								^*^Reference substance
	**3–4**	**5–6**	**7–8**	**9–10**	**11–12**	**13**	**Pupa**	**Adult**	**Whole egg**
**NEAA**									
Ala	52.2 ± 0.6^b^	53.9 ± 0.2^a^	52.1 ± 0.1^b^	51.3 ± 0.3^b^	49.4 ± 0.1^c^	46.4 ± 0.7^d^	44.3 ± 0.8^e^	36.2 ± 1.0^f^	6.67
Arg	41.0 ± 0.7^a^	40.5 ± 0.9^a^	38.6 ± 0.2^b^	36.9 ± 1.1^c^	36.2 ± 1.2^c^	33.3 ± 2.7^cd^	29.3 ± 1.3^d^	21.5 ± 1.1^e^	7.87
Asp	55.1 ± 2.4^ab^	55.7 ± 0.8^a^	53.9 ± 2.0^ab^	53.1 ± 1.3^b^	51.3 ± 0.9^bc^	50.1 ± 0.6^c^	48.5 ± 1.1^c^	41.1 ± 3.3^d^	12.7
Cys	7.7 ± 0.2^c^	8.9 ± 0.0^a^	8.1 ± 0.0^b^	7.2 ± 0.1^d^	6.3 ± 0.2^e^	5.5 ± 0.1^f^	4.6 ± 0.4^g^	3.2 ± 0.4^h^	3.85
Glu	69.9 ± 1.1^b^	71.9 ± 0.9^a^	70.2 ± 0.5^b^	68.4 ± 1.1^bc^	67.2 ± 0.6^c^	65.7 ± 0.6^d^	60.4 ± 0.9^e^	53.7 ± 2.7^f^	16.3
Gly	29.8 ± 0.3^c^	30.6 ± 0.2^b^	31.4 ± 0.1^a^	28.9 ± 0.7^c^	26.2 ± 0.9^d^	26.1 ± 0.3^d^	24.2 ± 0.6^e^	19.7 ± 1.0^f^	4.08
Pro	49.1 ± 1.2^a^	50.5 ± 0.8^a^	50.1 ± 1.0^a^	48.2 ± 3.2^ab^	44.2 ± 1.7^b^	40.4 ± 2.3^b^	35.1 ± 1.8^c^	31.4 ± 0.9^d^	5.6
Ser	26.9 ± 0.5^a^	27.1 ± 0.9^a^	28.0 ± 1.2^a^	27.1 ± 1.5^a^	24.3 ± 0.6^b^	23.4 ± 0.3^b^	20.9 ± 0.2^c^	16.8 ± 0.9^d^	9.19
Tyr	41.3 ± 0.3^c^	42.0 ± 0.2^b^	43.1 ± 0.1^a^	39.7 ± 0.1^d^	36.9 ± 0.8^e^	35.0 ± 1.8^e^	30.6 ± 0.8^f^	28.7 ± 1.4^f^	5.12
									
**EAA**									
His	29.9 ± 0.5^a^	28.1 ± 0.6^b^	27.9 ± 0.1^b^	26.1 ± 0.3^c^	24.4 ± 0.9^d^	22.4 ± 1.0^e^	19.0 ± 0.6^f^	16.4 ± 1.7^g^	2.83
Lys	33.3 ± 0.1^c^	36.9 ± 0.9^a^	36.1 ± 0.7^a^	34.1 ± 0.2^b^	32.1 ± 1.0^d^	32.3 ± 1.0^cd^	31.5 ± 1.4^d^	30.6 ± 1.9^d^	8.32
Thr	30.5 ± 1.9^a^	29.7 ± 1.7^ab^	28.7 ± 1.1^ab^	26.1 ± 2.0^b^	22.6 ± 0.7^c^	21.5 ± 1.2^cd^	20.3 ± 1.4^d^	19.1 ± 0.9^d^	5.94
Ile	27.5 ± 0.0^b^	27.9 ± 0.3^a^	26.1 ± 0.1^c^	24.2 ± 0.3^d^	21.5 ± 0.5^e^	19.7 ± 0.2^f^	16.1 ± 0.1^g^	13.2 ± 0.5^h^	6.16
Leu	35.9 ± 0.4^b^	36.6 ± 0.1^a^	35.1 ± 0.2^c^	33.3 ± 0.2^d^	32.4 ± 0.1^e^	29.7 ± 1.9^f^	22.1 ± 0.3^g^	19.1 ± 1.1^h^	10.5
Met	9.8 ± 0.1^a^	8.1 ± 0.0^b^	7.2 ± 0.0^c^	6.5 ± 0.0^e^	6.9 ± 0.1^d^	5.9 ± 0.9^de^	4.4 ± 0.1^f^	3.2 ± 0.1^g^	4.18
Phe	24.4 ± 0.2^a^	23.1 ± 0.0^b^	23.9 ± 0.4^a^	22.6 ± 0.1^c^	20.6 ± 0.9^d^	18.8 ± 0.1^e^	15.3 ± 0.5^f^	12.3 ± 1.0^g^	6.6
Trp	4.5 ± 0.1^a^	4.2 ± 0.0^b^	3.9 ± 0.2^c^	3.2 ± 0.1^d^	3.0 ± 0.2^d^	2.6 ± 0.0^e^	1.9 ± 0.1^f^	1.4 ± 0.3^g^	1.66
Val	39.5 ± 0.3^b^	40.4 ± 0.5^a^	40.7 ± 0.2^a^	38.6 ± 0.2^c^	35.1 ± 0.5^d^	34.4 ± 1.5^de^	32.1 ± 0.9^e^	25.4 ± 0.7^f^	7.34
EAAI	88.16	89.22	86.06	82.52	86.79	80.62	72.77	68.66	

* *The data of reference substances from the United States Department of Agriculture (USDA) (38); NEAA, non-essential amino acid; EAA, essential amino acid; EAAI, Essential Amino Acid Index. Data are presented as the mean ± SD. Means with different superscripts in column differ significantly (p ≤ 0.05). n = 3*.

**Figure 2:**
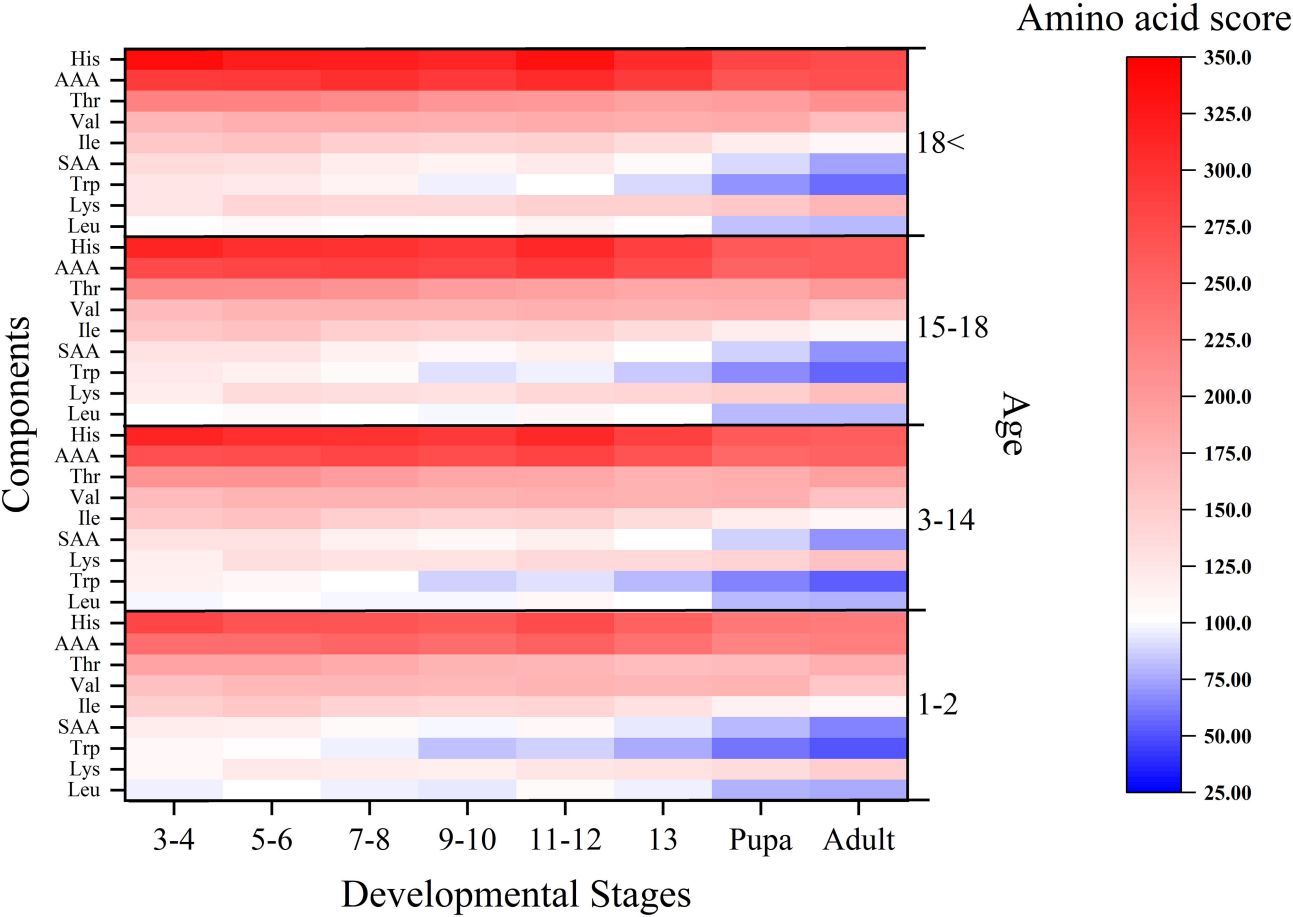
Amino acid scores at the different development stages of *Tenebrio molitor* for infants, children, adolescents and adults.

We obtained the amino acid scores of the yellow mealworms at different stages according to the amino acid scoring patterns for infants, children, adolescents, and adults from the 2007 WHO/FAO/UNU report (40). As shown in Figure 2, the yellow mealworms contained all phases of the restrictive amino acids such as tryptophan, isoleucine, sulfur-containing amino acids (SAA) and other aromatic amino acids (AAA), however, the amino acid score is declining with the development of yellow mealworms. Thus, the protein nutritional value of 13th instar yellow mealworms that insect farming chose usually is obviously inferior to that of the earlier instar larvae due to the deficiency of tryptophan, leucine and sulfur-containing amino acids.

The score of amino acids and the EAAI increased when mealworms were aged at instars 3–6, then decreased at instars 7–10, and then showed a slight rebound at instars 11–12. The amino acid scores of 13-instar larvae decreased continuously until the pupal and adult stages (Table 3, Figure 2). Compared with the growth curve of the yellow mealworms, the results were analyzed by combining with the study of Babu D E (45): 7–10 instars were the stage of rapid growth for yellow mealworms. The amino acids in the body of these yellow mealworms, especially in terms of the content of essential amino acids, were rapidly consumed by individuals as energy and raw materials, so the score of essential amino acids in the body and the EAAI value decreased during this period. Before and after 7–10 instars, the growth rate slowed down and the larvae entered a developmental slow period. The yellow mealworms reserved substances and energy for the later pupal stage, so the score of essential amino acids and EAAI increased.

### Selection of Harvest Time

To summarize, the final larvae, which are usually used as harvest options, scored the worst in nutritional estimates of protein compared to larvae with other instars. The amino acid contents of the young and middle-aged larvae were higher than that of the old mature larvae. The contents of all kinds of essential amino acids were also more in line with the requirement standard of essential amino acids recommended by WHO/FAO (40). On the other hand, the contents of chitin and fat increased with the development of mealworms, but humans cannot absorb and digest chitin. Furthermore, the fat rich in yellow mealworms accelerates the rancidity of the coarse processed products of yellow mealworms, which is not conducive to preservation (46). Thus, younger mealworms are easier to digest and preserve because they are lower in chitin and fat.

It must be admitted that the larvae with smaller harvest instars did not have the yield advantage. From another point of view, the advantage of the single weight of older larvae can make up for the disadvantage of low amino acid contents, but the feed utilization efficiency of insects will continue to decline with the aging of larvae. It can be inferred from the analysis in 3.2 that, under the condition of the same intake of feed, the growth rate slows down significantly and the biomass conversion ratio decreases significantly with the development of larvae. A large amount of feed is used to provide energy, and the rate of feed waste increases (47). In fact, instar growth was considered to be the most effective factor in reducing feed availability in mealworms (48). Therefore, it is not economical to insist on harvesting final instar larvae.

As a result, the earlier instars (the 9th to 10th instars in this study) are the better choices because of their nutritional value, preservation, feed cost, and other factors. Considering the cost and risk of feeding under various circumstances, the optimal harvest age of yellow mealworms still needs further study.

## Conclusion

The study was helpful in understanding the diversity of *T. molitor* at different developmental phases in terms of their growth curve, proportions of different nutritional ingredients, and amino acid composition. The study concluded that fat content is the highest in the pupal stage and chitin content is the highest in the adult stage. The protein content of *T. molitor* also decreased with the increase of instars, and the nutritional value of the proteins changed with the variation of the physiological status of the yellow mealworms. Furthermore, it was found that the protein yield and quality of the earlier stage of larvae are better. Due to the low chitin and fat content, easy digestion, utilization and preservation, better protein content and nutritional value, and higher feed utilization rate, a good harvest time could be when the larvae are still young. Combined with the yield, feed availability, nutritional value of protein, and other factors, 9th- to 10th-instar yellow mealworms would be a better choice for harvesting than the 13th-instar larvae. This study will provide reference for the improvement of mealworm production value and the selection of the best harvest time for other edible insects in the future.

## Data Availability Statement

The original contributions presented in the study are included in the article/supplementary material, further inquiries can be directed to the corresponding author/s.

## Author Contributions

DW conceived the project and provided lab condition and funding. XY and QH conducted the experiments and data collection. XY performed data analysis and wrote the original draft of the manuscript. All authors read to the manuscript and approved the submitted version.

## Funding

This study was supported by Innovation and Transformation Project for Agricultural Science and Technology of Shaanxi Province (NYKJ-2019-YL37).

## Conflict of Interest

The authors declare that the research was conducted in the absence of any commercial or financial relationships that could be construed as a potential conflict of interest.

## Publisher's Note

All claims expressed in this article are solely those of the authors and do not necessarily represent those of their affiliated organizations, or those of the publisher, the editors and the reviewers. Any product that may be evaluated in this article, or claim that may be made by its manufacturer, is not guaranteed or endorsed by the publisher.
